# Studies on membrane topology, N-glycosylation and functionality of SARS-CoV membrane protein

**DOI:** 10.1186/1743-422X-6-79

**Published:** 2009-06-18

**Authors:** Daniel Voß, Susanne Pfefferle, Christian Drosten, Lea Stevermann, Elisabetta Traggiai, Antonio Lanzavecchia, Stephan Becker

**Affiliations:** 1 Institute of Virology, Philipps-University Marburg, 35043 Marburg, Germany; 2 Robert Koch-Institute, 13353 Berlin, Germany; 3 Clinical Virology, Bernhard-Nocht Institute for Tropical Medicine, 20359 Hamburg; 4 University of Bonn Medical Centre, Institute of Virology, 53127 Bonn German, Germany; 5 Institute for Research in Biomedicine, CH 6500 Bellinzona, Switzerland

## Abstract

The glycosylated membrane protein M of the severe acute respiratory syndrome associated coronavirus (SARS-CoV) is the main structural component of the virion and mediates assembly and budding of viral particles. The membrane topology of SARS-CoV M and the functional significance of its N-glycosylation are not completely understood as is its interaction with the surface glycoprotein S. Using biochemical and immunofluorescence analyses we found that M consists of a short glycosylated N-terminal ectodomain, three transmembrane segments and a long, immunogenic C-terminal endodomain. Although the N-glycosylation site of M seems to be highly conserved between group 1 and 3 coronaviruses, studies using a recombinant SARS-CoV expressing a glycosylation-deficient M revealed that N-glycosylation of M neither influence the shape of the virions nor their infectivity in cell culture. Further functional analysis of truncated M proteins showed that the N-terminal 134 amino acids comprising the three transmembrane domains are sufficient to mediate accumulation of M in the Golgi complex and to enforce recruitment of the viral spike protein S to the sites of virus assembly and budding in the ERGIC.

## Background

Coronaviruses have a broad range of vertebrate hosts and usually cause mild respiratory diseases in humans and animals [1-3]. In March 2003 the world health organization issued a global alert about a severe partially fatal respiratory disease named severe acute respiratory syndrome (SARS). SARS originated in Southeast China, affected thousands and spread to many countries worldwide via international travel. Drastic quarantine measures and tight travel restrictions finally contained the SARS outbreak [4,5]. In parallel, the etiologic agent of the outbreak was identified with unprecedented speed and turned out to be a novel coronavirus with several distinguishing features to known coronaviruses [6-8]. The SARS outbreak showed drastically that coronaviruses can develop into highly pathogenic agents with the potential to threaten public health and global economy severely [9,10].

**Figure 1:**
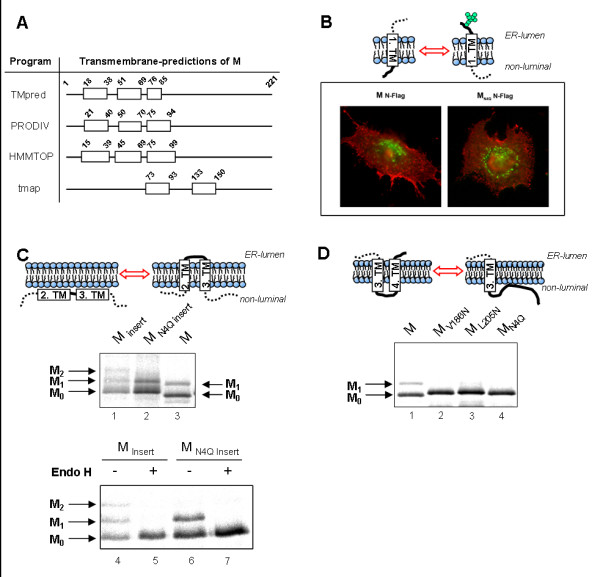
**Analysis of the membrane topology of SARS-CoV M**. A, *in silico *predictions of hydrophobic domains and potential transmembrane segments of M using various computer algorithms. Numbers in superscript refer to the position of the first and the last amino acids of potential transmembrane domains (white rectangles). B, Subconfluent Huh7 cells were transfected with plasmids encoding M (M_N-FLAG_) or a glycosylation-deficient M (M_N4QN-FLAG_) both N-terminally fused with a FLAG-peptide. Surface-staining (red fluorescence) and subsequent intracellular staining (green fluorescence) of M was performed 24 h posttransfection (p.t.) using a polyclonal α-FLAG and a fluorescence-labelled secondary antibody. C, M and the glycosylation mutants M_Insert _and M_N4Q __Insert _were in vitro translated in the presence of canine microsomal membranes and metabolically labelled with [^35^S] PROMIX (Promega). Resultant proteins were digested with Endo H. Membrane-bound proteins were pelleted and subjected to SDS-PAGE analysis. Radioactive signals were visualized using Bioimager analyser (BAS-1000; Fuji). M_0 _– non-glycosylated M; M_1 _– mono-glycosylated M; M_2 _– di-glycosylated M. D, M and the glycosylation mutants M_V186N _and M_L205N _were in vitro translated and analysed as described above.

The coronaviral membrane protein (M) is the most abundant protein in the viral envelope and fulfils pivotal functions in the viral life cycle. Besides mediating incorporation of the nucleocapsid into the newly formed virions, M recruits all other viral structural components to the ER-Golgi-intermediate compartment (ERGIC) where virus assembly and budding takes place [11-13]. While the topology of SARS-CoV M is yet unknown and *in silico *analyses of its topology revealed partially contradictory results (Fig. 1), previous publications showed that other coronaviral M proteins consist of three transmembrane domains with either an N-terminal ecto- and a C-terminal endodomain (N_exo_-C_endo_) or an N_exo_-C_exo _orientation [14-16].

SARS-CoV M is N-glycosylated at asparagine residue at position 4 and accumulates at steady state in the Golgi complex [17]. Whether glycosylation of M plays a role in its intracellular distribution and assembly of viral particles is not known. Immunofluorescence analyses demonstrated that M is mainly retained in the ERGIC [17,18].

In the present study we have investigated the topology of M and found that M consists of an N-terminal ectodomain containing the single N-glycosylation site, three transmembrane domains and a long cytosolic C-terminus. N-glycosylation is no prerequisite for accumulation of M in perinuclear regions. By using an infectious clone of SARS-CoV with a substitution in the N-glycosylation site of M, we observed that N-glycosylation is dispensable for assembly and infectivity of the virus. Furthermore, the hydrophobic N-terminus of M containing the three transmembrane domains is sufficient to recruit the SARS-CoV spike protein S to the budding compartment suggesting protein-protein-interaction between M and S via the transmembrane domains of M.

## Results

### Topology of SARS-CoV M

To predict membrane-spanning helices in SARS-CoV M, we performed hydrophobicity analyses and secondary structure predictions using several computer programs (Fig. 1A). Three in four algorithms predicted three transmembrane helices at similar positions. However, the tmap-program predicted only two hydrophobic domains between amino acids 73–93 and 133–150. Further *in silico *analyses also differed in the prediction of the topology of the N- and the C-terminus of M.

To analyze the membrane topology of M experimentally, we benefited from the fact that a fraction of recombinant FLAG-tagged M is detectable at the plasma membrane [17]. The FLAG epitope was fused to the N-terminus of M and the glycosylation-deficient mutant M_N4Q _and the accessibility of the FLAG Tag at the cell surface was analyzed by immunofluorescence of native cells expressing the two constructs. The experiments revealed that M_FLAG _as well as M_N4Q-FLAG _were detectable at the plasma membrane (Fig 1B). These results suggested that both M and M_N4Q _exhibit a N_exo_-orientation with the N-terminus facing the luminal or extracellular space (Fig. 1B, right graphic).

To further characterize the membrane integration of M, we introduced a novel glycosylation site (N-X-S) in the predicted intraluminal loop between hydrophobic domains 2 and 3 of the protein (Fig. 1C). To avoid a very close proximity of the glycosylation site and the ER membrane leading to sterical limitations of the oligosaccharide transferase in the ER lumen [19], we inserted 31 hydrophilic amino acids with a central N-glycosylation motif between amino acids 73 and 74 of M and M_N4Q _(named M_Insert; _M_N4Q Insert_). The *in vitro *expression of M_Insert _resulted in three protein bands (Fig. 1C, lanes 1 and 4) which could be attributed to non-, mono- and di-glycosylated forms of M by Endo H digest (Fig. 1C, lane 5). Expression of M_N4Q Insert _which contains only the novel glycosylation site between the potential transmembrane segments 2 and 3 resulted in two M-specific bands (Fig. 1C, lanes 2 and 6). Endo H digestion revealed that the upper band was glycosylated suggesting that the two forms correspond to non- and monoglycosylated M_N4Q Insert_. (Fig. 1C, lane 7). These results demonstrate that the linker-domain between the putative second and third hydrophobic domains faces the luminal site and the two hydrophobic stretches are probably incorporated in the lipid-layer. Therefore we hypothesize a membrane topology of M as depicted in the right graphic in Fig. 1C.

*In silico *analysis using the computer program tmap identified a hydrophobic stretch between amino acids 133 and 150 suggesting an additional transmembrane domain, which would direct the C-terminus of M into the lumen. (Fig. 1A). To analyse, whether parts of the C-terminus of SARS-CoV M are located in the cytoplasm, we constructed further glycosylation mutants of M carrying single N-glycosylation motifs at position 186 (M_V186N_) or 205 (M_L205N_) (Fig. 1D). *In vitro *translation of these constructs in the presence of microsomes revealed that neither M_V186N _nor M_L205N _were glycosylated (Fig. 1D, lanes 2 and 3) suggesting that the C-terminus of M is completely located in the cytoplasm (Fig. 1D, right graphic).

In summary, we could for the first time provide experimental support for a N_exo_-C_endo _topology of SARS-CoV M with a luminal N-terminus, three transmembrane segments and a long cytosolic C-terminus.

### Suppressed N-glycosylation of M does not impair protein accumulation in the Golgi complex nor the assembly and infectivity of a recombinant SARS-CoV

**Figure 2:**
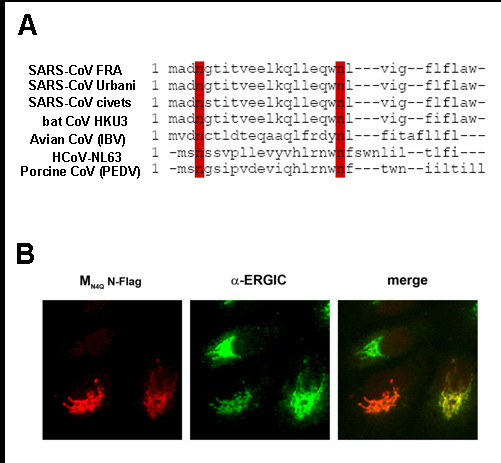
**N-glycosylation of coronviral M**. A, N-terminal amino acid sequences of M of selected coronaviruses were aligned and depicted using MacVector program. B, M and the glycosylation-deficient mutant M_N3Q _of HCoV-NL63 were in vitro translated and analyzed as described above. (Plasmid expressing HCoV-NL63 M protein was kindly provided by M. Müller, Bonn). C, Subconfluent Huh7 cells were transfected with plasmid encoding the glycosylation-deficient M (M_N4QN-FLAG_) using FuGENE according to the manufacturer's protocol. At 24 h p.t., cells were fixed, permeabilized and incubated with a polyclonal α-FLAG antibody and a rhodamine-coupled secondary antibody. ERGIC was stained using a monoclonal α-ERGIC primary antibody and a FITC-coupled secondary antibody. The merged image is shown on the right.

Amino acid sequence alignments demonstrated that the N-glycosylation motif at the extreme N-terminus of M is conserved between coronaviruses of group 1 and 3 and the newly emerged SARS-CoV (Fig. 2A). To confirm these *in silico *data the N-terminal N-glycosylation site of the homologue protein of another human pathogenic coronavirus, HCoV-NL63, was mutated which led to the loss of glycosylation completely as it is the case for SARS-CoV M (data not shown) [17]. These results induced the idea that N-glycosylation might be important for protein transport, protein-protein-interactions or other steps in the viral life cycle. Therefore, the intracellular distribution of the glycosylation-deficient mutant M_N4Q _of SARS-CoV was investigated by immunofluorescence analysis (Fig. 2B). The staining of the ER-Golgi intermediate compartment (ERGIC) and the recombinantly expressed M_N4Q _revealed a strong colocalization suggesting that the M_N4Q _as well as wild type M accumulates in the budding compartment ERGIC and that N-glycosylation has no influence on intracellular transport of M (Fig. 2B, right image)[17].

**Figure 3:**
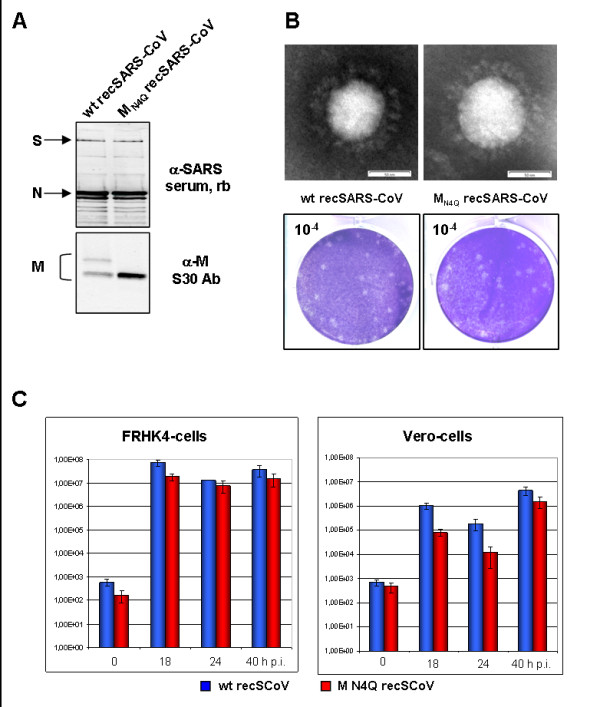
**Characterization of recombinant SARS-CoV expressing a glycosylation-deficient M**. A, Vero cells were infected with wild type recombinant SARS-CoV (wt rec SARS-CoV) or the mutant M_N4Q _recombinant SARS-CoV (M_N4Q _rec SARS-CoV) respectively with an moi of 3 pfu per cell. Cell lysates were harvested and subjected to Western blot analysis. Virus inactivation was achieved by treatment with 1% SDS and boiling of the samples. Proteins were separated by SDS-PAGE and blotted onto PVDF membranes. To detect viral structural proteins the membrane was incubated with a serum of an immunized rabbit or with the human monoclonal antibody S30. B, For electron microscopic analysis, Vero cells were infected with a MOI of 1 with wt rec SARS-CoV or M_N4Q _rec SARS-CoV, respectively. At 24 h p.i., viral particles were fixed, negatively stained and subjected to electron microscopic analysis.

Recently, a T7 promoter-driven infectious cDNA-clone of SARS-CoV was engineered [20]. To study N-glycosylation of M in the viral context, we applied this reverse genetic system and constructed a recombinant SARS-CoV (recSARS-CoV) expressing a glycosylation-deficient M. Expression of viral proteins was confirmed by western blot analysis using serum of a SARS-convalescent or the human monoclonal α-M antibody S30 [17]. The serum recognized the structural components spike- (S) and nucleocapsid- (N) protein in cell lysates of wt and mutant recSARS-CoV infected Vero-cells. The M specific antibody could indeed detect the unglycosylated and the N-glycosylated form of M in wt recSARS-CoV infected Vero-cells (Fig. 3A). However, in cells infected with M_N4Q _recSARS-CoV only the unglycosylated form of M was detected. These results provide evidence that we successfully rescued a replication-competent recSARS-CoV expressing a glycosylation-deficient M. To illustrate the morphology of wt as well as mutant recSARS-CoV, we performed electronmicroscopy analysis of purified virions demonstrating that both virions share a characteristic corona-solis like appearance (Fig. 3B, upper panel). By measuring several virions we observed that the average size of wt and mutant virions was the same.

To compare growth properties between wt recSARS-CoV and mutant recSARS-CoV, we first used plaque assays. As shown in Fig. 3B, the plaque size of the mutant virus was similar to wt virus presuming that the spread of the virus expressing a glycosylation-deficient M is not inhibited (Fig. 3B, lower panel). To further analyze the growth kinetics of both viruses, cell cultures were infected and the viral replication efficiency was assessed by calculating the number of viral genomes in the supernatant by real-time RT-PCR (Fig. 3C). In FRHK4 cells (fetal rhesus monkey kidney), wt recSARS-CoV (blue bars) as well as M_N4Q _recSARS-CoV (red bars) exhibited a considerable 10^6^-fold increase in viral genomes copies within 18 h p.i. and reached a plateau of approximately 10^7 ^viral genomes late in infection (40 h p.i.; Fig. 3C, left panel). These results were independent of the used cells since infection of Vero cells with both viruses resulted in similar amounts of viral particles late in infection (40 h p.i.; Fig. 3C, right panel). However, infection with M_N4Q _recSARS-CoV resulted in a 10-fold reduction of released genome copies early in infection compared to wt rec SARS-CoV. In general, both recombinant viruses possessed a delayed viral replication in infected Vero cells compared to FRHK4 cells.

In summary, we could provide evidence that a suppressed N-glycosylation of SARS-CoV M do not have a significant impact on protein transport and on assembly and replication of new virions in cell culture.

### The hydrophilic cytosolic tail of M exhibits an immunogenic epitope

**Figure 4:**
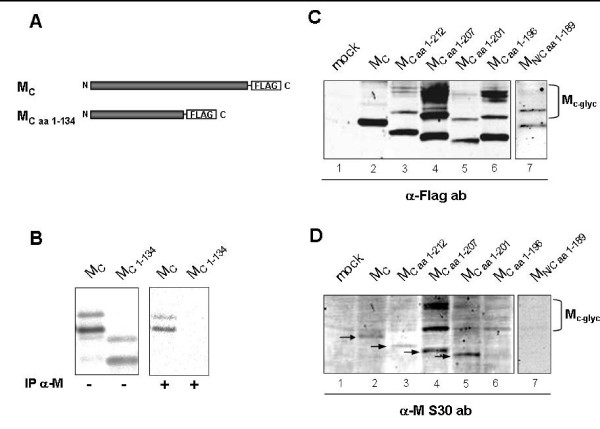
**Mapping the epitope of the human monoclonal α-M antibody S30**. A, An exemplary schematic representation of M mutants used in this study. A FLAG-epitope was fused to the C-terminus or in addition to the N-terminus. B, M_1–134 _was *in vitro *translated in the presence of [^35^S] PROMIX and subsequently immunoprecipitated using human monoclonal α-M antibody S30. The precipitates were subjected to SDS-PAGE and radioactive signals were visualized by autoradiography. C and D, BHK-T7 cells were transfected with plasmids encoding various C-terminally truncated M mutants. At 24 h p.t., cells were lysed, sonicated and directly subjected to SDS-PAGE analysis followed by immunoblotting using polyclonal α-FLAG and human monoclonal α-M antibody S30 simultaneously. The proteins were visualized using an AF680-coupled α-rabbit and an IRDye800-coupled α-human antibody and the Odyssey™ Infrared Imaging System. M_c-g_, complex glycosylated form of M. Arrowheads indicate the specific protein bands detected by α-M S30 antibody.

Previously, we could identify the M-specific antibody S30 synthesized by Epstein Barr virus-immortalized B-lymphocytes of a SARS convalescent [21]. To map the epitope of this unique M-specific human monoclonal antibody, we constructed FLAG-tagged mutants with truncations in the C-terminal part of M. The respective mutants were expressed in BHK-T7 cells and employed in immunoblot and immunoprecipitation analysis (Fig. 4A and 4B). M and M_1–134 _were metabolically labelled during *in vitro *translation and precipitated using S30. While M was readily precipitated by S30, M_1–134 _was not (Fig. 4B). To narrow down the epitope recognized by S30 in the C-terminus of M, FLAG-tagged mutants of M were constructed which carried small truncations in the absolute C-terminus (Fig. 4C and 4D). Mutants were expressed in BHK-T7 cells and cell lysates were subjected to western blot analysis. While all mutants were readily detected by an anti-FLAG antibody, only mutants consisting of at least 201 amino acids could be detected by S30 (Fig. 4D, lanes 5 and 6). Unexpectedly, mutant M_1–196 _comprising of 196 amino acids migrated slightly slower than the mutant consisting of 201 amino acids. Since the sequence of the M_1–196 _had been verified like all other constructs, the reason for the abnormal behaviour in SDS-PAGE remained unknown.

We were not able to detect the truncated mutant M_1–189_. Transient expression of M_N/C1-189 _possessing a FLAG-tag at the N- and the C-terminus and subsequent Western blot analysis revealed the expression of the mutant, which was not detected by the S30 antibody. Taken together, it appears that S30 recognizes a distinct linear epitope located at the extreme C-terminus of M around amino acid residues 196 and 201.

### The hydrophobic N-terminus of M is sufficient to recruit M in the Golgi-complex and to retain S in perinuclear regions

**Figure 5:**
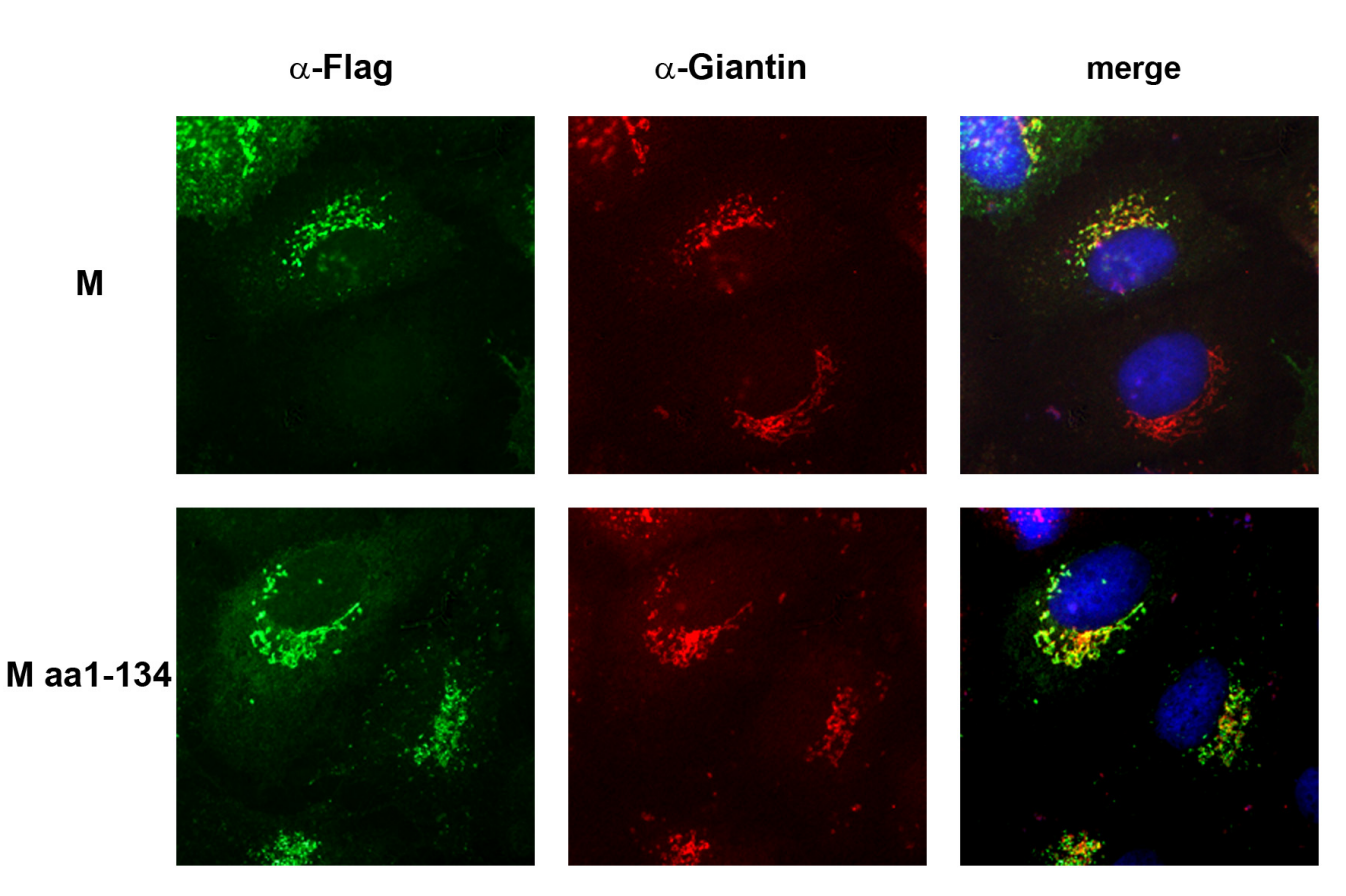
**Intracellular distribution of M and M_1–134_**. Huh7 cells were transfected with plasmids encoding M or M_1–134 _as described in Fig. 2. At 24 h p.t., cells were fixed, permeabilized and incubated with a polyclonal α-FLAG and a monoclonal α-Giantin antibody followed by a FITC-coupled α-rabbit and a rhodamine-coupled α-mouse antibody. The merged images are shown on the right hand side, respective proteins are given on the left hand side.

M is concentrated in the Golgi compartment but the molecular determinants for the intracellular distribution are not understood. Therefore, we analyzed the intracellular distribution of the C-terminal truncated mutant M_1–134 _by immunofluorescence (Fig. 5). As expected, full-length M accumulated in Golgi complex of transiently transfected Huh7-cells (Fig. 5, upper row). The recombinant expression of M_1–134 _showed strong colocalization with Golgi-marker as well suggesting that the hydrophobic N-terminus of M constitutes a Golgi-retention signal (Fig. 5, lower row).

**Figure 6:**
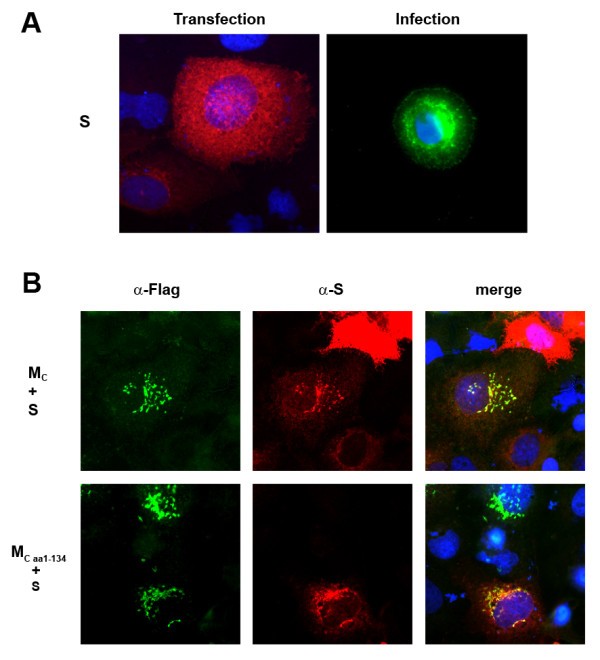
**Coexpression of M and S**. A, Huh7 cells were transfected with plasmid as described in Fig. 2. At 24 h p.t., cells were fixed, permeabilized and viral proteins were stained with a rabbit polyclonal α-S serum and a rhodamine-coupled α-rabbit secondary antibody. SARS-CoV (strain Frankfurt) infected Vero cells were fixed, permeabilized and incubated with the same antibodies as described above. B, Huh7 cells coexpressing M_C _or the truncated form M_1–134 _together with S were fixed, permeabilized and incubated with a rabbit α-S serum and a mouse monoclonal α-FLAG antibody followed by a FITC-coupled α-mouse and a rhodamine-coupled α-rabbit secondary antibody. The merged images are shown on the right hand side and respective, coexpressed proteins are given on the left hand side of the panels.

The recruitment of all coronaviral structural components in the perinuclear region is crucial for viral assembly and budding. However, recombinant spike protein S was transported to the plasma membrane upon single expression (Fig. 6A, left panel), while S in SARS-CoV infected cells was localized mainly to the perinuclear region (Fig. 6A, right panel). Thus, we explored whether M influenced intracellular localization of S. To this end, M and S were coexpressed and their respective intracellular localization was analyzed by immunofluorescence (Fig. 6B, upper row). We found that in the presence of M, S was recruited to cellular compartments where SARS-CoV budding takes place. Replacing M by the mutant containing only the hydrophobic N-terminal part of the protein, M_1–134_, we observed that S is still found in the perinuclear region and plasma membrane transport is prevented (Fig. 6B, lower row). In summary this experiment showed that coexpression with at least the first 134 amino acids of M induced a redistribution of the surface protein S to the perinuclear region which is consistent with the idea that M recruited S to site of assembly and budding of SARS-CoV.

## Discussion

M proteins of coronaviruses with low pathogenic potential for humans are quite well understood in terms of synthesis, transport and function. M protein of the highly pathogenic SARS-CoV shows little amino acid sequence homology to other coronaviruses which makes predictions difficult [22]. In this report, we analyzed SARS-CoV M with respect to membrane topology, N-glycosylation and functional interaction with the surface protein S.

### Membrane topology and functional analysis of M

**Figure 7:**
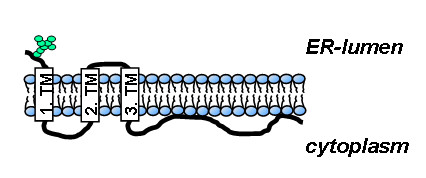
**Membrane topology model of SARS-CoV M protein**.

As shown previously, SARS-CoV M acquires one oligosaccharide side chain at asparagine residue 4 suggesting a luminal orientation of the N-terminus [17]. This result was supported and extended in the present manuscript by immunofluorescence analyses demonstrating that even the glycosylation-deficient M exhibits an N-terminal ectodomain. The topology of the hydrophobic stretches in M was determined using artificially incorporated N-glycosylation sites postulating the presence of three transmembrane domains and a long cytosolic C-terminus. The topology is different to other coronaviruses e.g. the transmissible gastroenteritis virus (TGEV) M [15]. The presented membrane topology model of SARS-CoV M presumes a "N_exo_-C_endo_"-orientation (Fig. 7).

We further identified an immunogenic B-cell epitope at the very C-terminus of M. These results supported the observation that the cytosolic tail and in particular the amino acids between position 195 and 210 of M can serve as an efficient immunogen in antibody production (He et al., 2005; Imgenex 5125A). Obviously, M contains several immunogenic epitopes since sera from SARS patients recognized additional epitopes located in the C- and N-terminal region of M as well [23,24]. Our attempts to generate antibodies to peptides representing the absolute N-terminus of M, however, were unsuccessful (not shown).

Presented functional analyses showed that the first 134 amino acids of SARS-CoV M comprising the three transmembrane domains are crucial for accumulation of M in the Golgi-complex. Similar findings have been reported for infectious bronchitis virus (IBV), which suggested retention signals in the first transmembrane domain of M [25]. An additional retention signal located in the cytosolic tail is postulated for MHV M [26]. Whereas M of MHV and IBV seem to be retained completely in perinuclear regions [11], M of TGEV, of the feline coronavirus (FCoV) and of SARS-CoV are also exposed at the plasma membrane [17,27,28].

### N-glycosylation of SARS-CoV M

Detailed analyses revealed that accumulation of M beyond the budding compartment in the Golgi-complex is a common feature of all investigated coronaviruses [11,17,29-31]. In this study we observed that N-glycosylation had no impact on the intracellular distribution of recombinant SARS-CoV M. By utilizing a novel reverse genetic system of SARS-CoV we also did not observe any significant influence of N-glycosylation of M in terms of viability, assembly and budding efficiency and infectivity of recombinant SARS-CoV in cell culture [20]. Supportive for these results are data by de Haan et al., showing that O-glycosylation of murine hepatitis virus (MHV) M is dispensable for assembly and spread of the virus [32,33]. Most likely the conserved glycosylation of M serves other functions. Previous studies on TGEV revealed an influence of glycosylation of M on interferon induction [34]. In the same direction point experiments in which an artificially introduced N-glycosylation in MHV M caused a strong interferon-α and -β induction while glycosylation-deficient MHV M is a poor interferon-α inducer [32].

### Influence of M on the intracellular distribution of S

Surface spikes composed of the SARS-CoV glycoprotein S confer the name-giving shape of coronaviral particles in electron microscopy analysis [35]. The spikes coat the viral envelope and facilitate interaction of the virus with the cellular receptor and subsequent fusion with the plasma membrane [36-40]. Hence, incorporation of S into the viral envelope is essential for infectivity of released progeny virions [41-43]. A retention signal located at the cytosolic C-terminus of most coronaviral S proteins prevents surface expression of S [44,45]. However, we and others could observe that upon single expression, S of SARS-CoV is detectable at the plasma membrane [36,45,46]. Thus, the question arose whether interaction with other viral proteins supported retention of S. Previous studies on MHV showed an interaction between M and S and emphasized the impact of this interaction on viral morphogenesis [12,42,43,47]. Here, we observed that M suppressed the surface transport of S and may thus functionally support the ER retrieval signal identified by McBride and Machamer [46]. Essential for the retrieval of S was the N-terminus of M.

## Conclusion

In summary, by using N-glycosylation mutants and subsequent Endo H digest we could establish a membrane topology model of SARS-CoV M with three transmembrane domains and a N_exo_-C_endo_-orientation. N-glycosylation of M is neither essential for transport and topology of M nor for its function in replication of recombinant SARS-CoV. Further *in vivo *studies using the generated N-glycosylation-deficient SARS-CoV are necessary to elucidate the role of conserved N-glycosylation during immune response of the infected host. Finally, our data revealed that the N-terminal part of M induces the retrieval of S to the ERGIC.

## Materials and methods

### Cells and Viruses

BSR T7/5 cells (BHK-21 cell clone), which constitutively expressed T7 RNA polymerase, were cultured as described by Bucholz *et al*. [48]. Monolayer cultures of the human hepatoma cell line Huh7 and the African green monkey kidney cell line Vero were grown at 37°C and 5% CO_2 _in Dulbecco's modified Eagles Medium (DMEM, Gibco) supplemented with 10% fetal calf serum (FCS) 100 U/ml penicillin, and 0.1 mg/ml streptomycin. For the infection of Vero cells SARS-CoV (Frankfurt strain; accession no. AY310120) was used at a multiplicity of infection of approximately 0.1.

### Molecular Cloning of M and Glycosylation-Mutants of M

For expression of M and the glycosylation-deficient M N4Q, the respective genes were cloned under the control of the T7 RNA polymerase promoter into the pTM1 plasmid as described previously (Voss et al., 2006). Cloning of the glycosylation mutants M_V186N _and M_L205N _was performed using QuickChange site-directed mutagenesis kit (Stratagene) to substitute valine and leucine by asparagine at the indicated amino acid positions. The construction of pTM1-M_Insert _and pTM1-M_N4Q__Insert _was performed by consecutive PCR-steps and subsequent cloning into pTM1 vector. First, fragments encoding amino acids 1–73 and 74–221 of M were amplified using pTM1-M or pTM1-M_N4Q _as template and the following primers: M_1–73 _(5'-CGGAATTCATGGCAGACAACGGTACTATTACCG-3' and 5'-acggtagcggttgtatgcgtaattaattctgtagacaacagc-3') and M_74–221_(5'-gacgatgacaagccaaaagagtgggtgactggcgggattgcg-3' and 5'-CGAGGATCCTTACTGTACTAGCAA AGCAATATTG-3'). In addition, a fragment encoding a stretch of 31 hydrophilic amino acids with a central N-glycosylation site was amplified using the partially complementary oligonucleotides 5'-TACGCATACAACCGCTACCGTATTGGAAACTATAAATTAAATACAACTATTAAGGACCTGGACTACAAGGAC-3' and 5'-CTCTTTTGGCTTGTCATCGTCGTCCTTGTAGTCCAGGTCCTTAATAGTTGTATTTAATTTATAGTTTCC-3'. The three resulting PCR fragments were fused by recombinant PCR leading to M_Insert _or M_N4Q Insert _respectively and cloned into the EcoRI and BamHI restriction sites of pTM1 vector. All constructs were verified by restriction analysis and sequencing.

### Molecular Cloning of M Truncation Mutants

DNA fragments encoding the C-terminally truncated M mutants were generated by PCR with pTM1-M as template and the following primers: M_1–107 _(5'- CCGGAATTCATGGACTACAAGGACGACGATGACAAGGCAGACAACGGTACTATTACCGTTG-3' and 5'-CGAGGATCCTTATGAGCGGGTACGAGC-3'); M_1–134 _(5'-CGGAATTCATGGCAGACAACGGTACTATTACCG-3' and 5'-CGAGGATCCTTACTTGTCATCGTCGTCCTTGTAGTCTTCCATGAGCGGTCTGGTCAC-3'); M_1–159 _(5'-CGGAATTCATGGCAGACAACGGTACTATTACCG-3' and 5'-CGAGGATCCTTACTTGTCATCGTCGTCCTTGTAGTCGTCACAGCGCCCTAGGGAGTG-3'); M_1–189 _(5'-CGGAATTCATGGCAGACAACGGTACTATTACCG-3' and 5'-CGAGGATCCTTACTTGTCATCGTCGTCCTTGTAGTCATCAGTGCCTACACGCTGCGAC-3'); M_N/C1-189 _(5'-CCGGAATTCATGGACTACAAGGACGACGATGACAAGGCAGACAACGGTACTATTACCGTTG-3' and 5'-CGAGGATCCTTACTTGTCATCGTCGTCCTTGTAGTCATCAGTGCCTACACGCTGCGAC-3'); M_1–196 _(5'-CGGAATTCATGGCAGACAACGGTACTATTACCG-3' and 5'-CGAGGATCCTTACTTGTCATCGTCGTCCTTGTAGTAGTTGTATGCAGCAAAACCTGAATCAGTGCC-3'); M_1–201 _(5'- CGGAATTCATGGCAGACAACGGTACTATTACCG-3' and 5'-CGAGGATCCTTACTTGTCATCGTCGTCCTTGTAGTCTCCAATACGGTAGCGGTTGTATGCAGC-3'); M_1–207 _(5'-CGGAATTCATGGCAGACAACGGTACTATTACCG-3' and 5'-CGAGGATCCTTACTTGTCATCGTCGTCCTTGTAGTCTGTATTTAATTTATAGTTTCCAATACGGTAGCGG-3'); M_1–212 _(5'-CGGAATTCATGGCAGACAACGGTACTATTACCG-3' and 5'-CGAGGATCCTTACTTGTCATCGTCGTCCTTGTAGTCGCTACCGGCGTGGTCTGTATTTAATTTATAG-3'). The fragments were cloned into the EcoRI and BamHI cleavage sites of pTM1. The integrities of all constructs were verified by restriction analysis and sequencing.

### Indirect Immunofluorescence Analysis

To perform surface staining of transiently transfected Huh7 cells, cells were grown on glass cover slides and transfected with the plasmids indicated in figure legends as described previously (Voss et al., 2006). At 24 h pt, cells were put on ice and successively incubated with a polyclonal α-FLAG (Sigma) and a secondary rhodamine-coupled α-rabbit antibody (Dianova), respectively. Intracellular staining was achieved by subsequent fixation with 4% PFA/DMEM for 15 min at room temperature, permeabilization with 0.1% Triton X-100 in PBS for 10 min and additional incubation for 1 h with a polyclonal α-FLAG and a FITC-coupled secondary α-rabbit antibody (Dianova) at room temperature. To characterize the influence of M on the intracellular transport of S, subconfluent Huh7 cells were co-transfected with pTM1-M_C-FLAG _or pTM1-M_1–134 _and pcDNA.3.1-S (kindly provided by M. Eickmann, Marburg) using FuGENE (Roche) according to the manufactor's protocol. In addition, a plasmid was cotransfected encoding the T7 DNA-dependent RNA-polymerase supporting the expression of the M mutants. At 24 h p.t., the cells were fixed with 4% PFA/DMEM for 15 min and permeabilized for 10 min with 0.1% Triton X-100. Then, unspecific binding sites were blocked using 3% BSA/PBS before the protein of interest was stained using rabbit polyclonal α-FLAG (Sigma) and/or a human monoclonal α-S antibody [21], respectively. The secondary antibodies used were donkey α-rabbit FITC-coupled and α-human rhodamine-coupled antibody (Dianova). All steps were performed at room temperature. Fluorescence images were taken with fluorescence microscope (Zeiss Axiovert 200M).

### Infectious cDNA-clone of SARS-CoV

Engineering of a T7-driven cDNA clone of SARS-CoV has been published previously [20]. Briefly, the viral genome of SARS-CoV (Frankfurt 1 isolate) was subdivided into seven fragments by RT-PCR and subsequently cloned into pCR2.1 (Invitrogen) or pSMART vectors (Lucigen) using appropriate restriction enzymes. In order to achieve a full length SARS-CoV cDNA clone the present constructs were subjected to further restriction/ligation steps into pBelo vectors finally assembling a pBelo-SARS clone under the control of the T7 promoter. The full-length BAC-constructs were linearized, *in vitro *transcribed and electroporated in BHK-21 cells using GenePulser (Biorad). Harvest and purification of infectious virus was done under BSL4-conditions. According to this approach, we constructed a cDNA-subclone with a substitution in the N-glycosylation site of M by site-directed mutagenesis (Quick change, Stratagene) using wild type pSMART-F as template. Subsequent cloning steps and the rescue of a recombinant SARS-CoV expressing glycosylation-deficient M was performed as described above for wild type recombinant SARS-CoV.

### Growth kinetics of recombinant viruses using real time RT-PCR

Vero or FRHK4 cells were infected with plaque-purified wt recSARS-CoV or M_N4Q _recSARS-CoV with a MOI of 0.05, respectively. At the indicated time points, viral RNA was isolated using QIAamp viral RNA kit according to the manufactor's protocol and subjected to real-time RT-PCR. To monitor viral replication, the viral polymerase gene was amplified using RT PCR [6]. Fluorescence signals were read by LightCycler^® ^(Roche).

### Electronmicroscopy Analysis

Negative staining of purified viral particles was carried out as described elsewhere [22,49].

### Amino acid sequence alignments

*In silico *protein analysis were performed with the following programs: TMpred, PRODIV, HMMTOP, and tmap.

### Other Methods

*in vitro *translation and subsequent Endo H-digest or immunoprecipitation analyses as well as transient transfection of BHK-T7 cells and Western blot analyses were carried out as described previously [17].

## Abbreviations

M: membrane protein; SARS: severe acute respiratory syndrome; SARS-CoV: SARS-Coronavirus; TGEV: transmissible gastroenteritis virus; S: spike protein; C-terminus: carboxyl-terminus; N-terminus: amino-terminus; TM: transmembrane domain; ER: endoplasmic reticulum; ERGIC: ER-Golgi-intermediate compartment.

## Competing interests

The authors declare that they have no competing interests.

## Authors' contributions

DV designed the study, conducted the experiments and wrote the manuscript. DV and SP carried out the experiments in BSL-4 facility. LS constructed mutants and helped to carry out the biochemical studies. CD, ET and AL contributed materials and/or critically revised the manuscript. SB, designed and coordinated the study, participated in the analysis of the results and drafted the manuscript. All authors read and approved the final manuscript.
